# Inhibition and Interactions of *Campylobacter jejuni* from Broiler Chicken Houses with Organic Acids

**DOI:** 10.3390/microorganisms7080223

**Published:** 2019-07-30

**Authors:** Ross C. Beier, J. Allen Byrd, Denise Caldwell, Kathleen Andrews, Tawni L. Crippen, Robin C. Anderson, David J. Nisbet

**Affiliations:** United States Department of Agriculture, Agricultural Research Service, Southern Plains Agricultural Research Center, Food and Feed Safety Research Unit, College Station, TX 77845-4988, USA

**Keywords:** acetic acid, *Campylobacter jejuni*, citric acid, formic acid, l-lactic acid, molar minimum inhibitory concentrations (MIC_M_s), organic acids, propionic acid, broiler chickens, susceptibility

## Abstract

*Campylobacter jejuni* is a bacterium that causes major diarrheal disease worldwide and is also one of the top five foodborne pathogens encountered in the United States. Poultry is a major source of *C. jejuni*, and a high-risk factor for contracting campylobacteriosis. Organic acids are used in the United States during food animal processing for removal of bacterial contamination from animal carcasses. Six organic acids were evaluated in inhibition studies of 96 *C. jejuni* strains obtained from shoe covers used in broiler chicken houses at different poultry farms in several states by determining the susceptibilities of the *C. jejuni* strains, along with the pH values at the molar minimum inhibitory concentrations (MIC_M_s). The undissociated and dissociated organic acid concentrations were calculated at the MIC_M_s with the Henderson-Hasselbalch equation. The results for the 96 *C. jejuni* strains were treated similarly for each different organic acid. *Campylobacter jejuni* inhibition did correlate with the dissociated organic acids, but did not correlate with pH or with the undissociated organic acids. When the concentrations of dissociated organic acids decreased, the *C. jejuni* strains were not disinfected. A carcass wash using organic acids should have the concentration of dissociated acid species carefully controlled. It is suggested to maintain a dissociated acid concentration for propionic, l-lactic, formic, citric, butyric, and acetic acids at 24, 40, 36, 21, 23, and 25 mM, respectively, and at these dissociated organic acid levels an acid wash would be expected to remove or inhibit 97% or more of the *C. jejuni* bacteria studied here. However, studies must be undertaken to confirm that the suggested concentrations of dissociated organic acids are adequate to remove *C. jejuni* bacteria in the field vs. the laboratory. Due to propionate, l-lactate, formate, butyrate, and acetate being utilized by *C. jejuni*, these organic acids may not be appropriate for use as a carcass wash to remove *C. jejuni* surface contamination. Of all tested organic acids, dissociated citric acid was the most efficient at inhibiting *C. jejuni*.

## 1. Introduction

*Campylobacter jejuni* is a bacterial organism that causes gastroenteritis worldwide [1,2,3,4,5,6]. *Campylobacter jejuni* are Gram-negative bacteria that are non-spore-forming [7] and are known to be a major cause of diarrheal disease in humans [8]. *Campylobacter* species are within the top five of the 31 major foodborne pathogens in the United States, and the Centers for Disease Control and Prevention (CDC) has estimated that each year, *Campylobacter* species cause 845,024 illnesses, 8,463 hospitalizations, and 76 deaths in the U.S. [9,10]. The two main *Campylobacter* species usually associated with human foodborne illnesses worldwide are *C. jejuni* and *C. coli* [2,11,12,13]. These two bacteria have high DNA homology [14], with related or identical antigens [15]. The most bacterial foodborne illnesses in the United States in 2016 were a result of *Campylobacter* and *Salmonella*, as described by the CDC [16]. However, most human *Campylobacter* infections are caused by *C. jejuni* [2,4,8,17]. Poultry have been found to be a major source of *C. jejuni* exposure, and consuming poultry food products is recognized as a high-risk factor for contracting campylobacteriosis [18,19,20,21,22,23]. *Campylobacter* infections may be fatal among young children, the elderly, and immunosuppressed patients (HIV patients) [6,24]. In particular, the incidence of *Campylobacter* infections in children <2 years of age in Israel has increased over the last few years to become the highest among industrialized countries [23]. A *C. jejuni* infective dose of only about 500–800 bacteria are required for infection [25,26].

New strategies to control animal-derived foodborne pathogens from the farm-to-fork must be developed to prevent illness due to these pathogens [27]. An important step used in the United States in the meat processing plant is a surface animal carcass wash containing organic acids (OAs) to remove bacterial contamination. Some short-chain OAs have been used to remove *C. jejuni* from avian carcasses in poultry processing plants [28,29,30,31], and studies using OAs have been conducted to remove or reduce *C. jejuni* populations in live birds during grow-out and preslaughter feed withdrawal [31,32,33,34,35,36]. The remaining bacteria found on carcasses in processing plants may later proliferate and be found on the processed meat, and meat marination studies have also been conducted using OAs to remove foodborne pathogens on the resulting processed chicken meat [37].

The mechanism of OA bacterial inhibition has been traditionally assumed to be mainly dependent on pH [38] or the undissociated form of the OAs [39,40,41,42,43,44], which are thought to penetrate the lipid membrane. However, the specific mechanism(s) by which OAs inhibit bacteria are not known [38,45,46,47]. In previous studies designed to evaluate foodborne pathogen interactions with OAs, molar units for concentration were used for the minimum inhibitory concentrations (MIC_M_s) of the OAs when comparing pH, and undissociated and dissociated OA data, because it results in an accurate analysis between MIC results of OAs with different molecular weights [48]. Our previous studies evaluated *Escherichia coli* O157:H7 [48], non-O157 Shiga toxin-producing *E. coli* (non-O157 STECs) [49], *Salmonella* enterica serovars [50], vancomycin-resistant *Enterococcus faecium* (VRE) [51], and *C. coli* [52] against OAs. These studies against OAs showed no correlation between pH or the undissociated OAs and bacterial MIC_M_s. However, the dissociated OAs did show a close correlation with the MIC_M_s, and the disintegration of a bacterial LPS layer has been demonstrated by a dissociated OA [53]. It was observed in previous research that bacteria would be expected to escape disinfection if the concentration of the dissociated OAs decreased below optimum levels [48,49,50,51,52].

Here, we studied the interactions of 96 *C. jejuni* strains isolated from shoe covers used in broiler chicken houses at different poultry farms in several different states with a number of OAs. Susceptibility studies were conducted with the 96 *C. jejuni* strains against the OAs, propionic, l-lactic, formic, citric, butyric, and acetic acid, and the pH was determined at the MICs. Calculations of the concentrations of undissociated and dissociated OAs at the MIC_M_s were undertaken. The pH and concentrations of the undissociated and dissociated OA species are compared at the MIC_M_s for all *C. jejuni* strains and show a good association between the dissociated OA species and bacterial inhibition.

## 2. Materials and Methods

### 2.1. *Campylobacter jejuni*

*Campylobacter jejuni* strains (*n* = 96) were isolated from broiler chicken houses at different poultry farms in several different states. Two shoe covers (97041-234, VWR, Missouri City, TX, USA) were placed into an autoclavable plastic container with 100 mL of 2× skim milk (232100, Difco™, Becton, Dickinson and Company, Sparks, MD, USA) and autoclaved for 15 min at 121 °C. Four pairs of shoe covers were used for each chicken house. The shoe covers were placed over boots and used as swabs to collect bacteria from the litter on the chicken house floor. The shoe covers were worn while walking ½ the length of the house on one side, and then they were removed and replaced with two fresh shoe covers. When reaching the end of the house, this process was repeated for the walk back to the front of the house. The four pairs of exposed shoe covers were packed in an ice chest and shipped to the laboratory. After arrival at the laboratory, 90 mL of buffered peptone water (212367, BBL™, Becton, Dickinson and Company, Sparks, MD, USA) was added to each pair of shoe covers and shaken. Samples were subjected to direct plating (one plate directly from each sample) and one from a 1:10 peptone water dilution (for a total of two plates) for enumeration on Campy-cefex agar (C-C) plates [54], and were incubated at 42 °C in a microaerobic environment (5% O_2_, 10% CO_2,_ 85% N_2_) for 48 h. Additionally, 5 mL of each peptone water sample was added to 5 mL of 2× Bolton enrichment broth [54] and incubated at 42 °C overnight. The samples were then plated onto C-C plates and incubated at 42 °C in the microaerobic environment for 48 h. Five colonies from each positive C-C plate were isolated and placed on trypticase soy agar w/5% sheep blood BD BBL™ Stacker™ (TSA) plates (#221261, Becton, Dickinson and Company, Sparks, MD, USA) in an anaerobic chamber (5% H_2_, 5% CO_2_, 90% N_2_), and then transferred to Brucella Broth (211088, BBL™, Becton, Dickinson and Company, Sparks, MD, USA) containing 20% glycerol (GX0185-5, EM Science, Gibbstown, NJ, USA) and placed at −80 °C for storage. Samples were also sent to the National Antimicrobial Resistance Monitoring System (NARMS) for Sensititre™ antimicrobial susceptibility testing.

### 2.2. Susceptibility Testing of *C. jejuni* Strains with Organic Acids

The *C. jejuni* OA MICs were obtained by using broth microdilution testing for fastidious bacteria, as described by the Clinical and Laboratory Standards Institute (CLSI) [55] and the TREK Diagnostic Systems published method for determining susceptibility using *Campylobacter* sensititre plates [56]. The susceptibility studies carried out here required *Campylobacter* incubation at 42 °C for 48 h because some strains did not sufficiently grow in 24 h, as was also observed in an earlier *Campylobacter coli* study [52]. Briefly, 50 μL of cation-adjusted Mueller-Hinton broth w/TES w/Lysed horse blood (Remel, Lenexa, KS, USA) was added to wells 2–12 of a 96-well U-bottom Greiner bio-one microplate (#650161, VWR International, LLC, Bridgeport, NJ, USA), and 50 μL of each standard OA solution was added to wells 1 and 2, and the solution in well 2 was further diluted 1:2 across the microplate through column 11, and the wells in column 12 were the positive controls [52]. *Campylobacter jejuni* colonies were then selected from TSA plates, and diluted into tubes containing 5 mL cation-adjusted Mueller-Hinton broth w/TES (Remel, Lenexa, KS, USA) to be equivalent to a 0.5 McFarland standard using a TREK Diagnostic Systems Nephelometer (East Grinsted, UK). Because the wells in the organic acid susceptibility plates contain a final volume of 100 μL, 200 μL of the 0.5 McFarland suspension was then added to tubes that contained 11 mL Mueller-Hinton broth w/TES w/Lysed horse blood, resulting in 1 × 10^6^ CFU/mL of each bacterial isolate, as previously described [52]. The horse blood bacterial solution (50 μL/well) was added to each well of the 96-well plates. A perforated plastic adhesive cover sheet (YG522EA, Remel) was placed over the bacteria-loaded microplates, and the covered plates were placed in the BD GasPak™ EZ incubation containers (Becton, Dickinson and Company, Sparks, MD, USA). Sachets (BD #260680) were enclosed in the EZ containers according to the manufacturer’s instructions, along with a 100 mL beaker half full of water to provide a moisturized atmosphere, and the containers were closed. This system provided a microaerobic atmosphere, where they were incubated for 48 h at 42 °C. The microplate well containing the smallest OA concentration that had no visible bacterial growth was considered the minimum inhibitory concentration (MIC) [57], as observed on a SensiTouch imaging system (TREK Diagnostic Systems Ltd., East Grinsted, UK). *Campylobacter jejuni* ATCC 33560 was run along with each test as the control organism. The results obtained here for ATCC 33560 were compared with results obtained from testing these same OAs against the control organism *Escherichia coli* ATCC 25922 in aerobic conditions, as ATCC 25922 was used as the control organism during all previous aerobic OA susceptibility testing studies [48,49,50,51,58,59,60,61].

The concentrations of OAs tested were equivalent to those previously used against *C. coli* [49]: propionic acid, 32,768–32 μg/mL; formic acid, 16,384–16 μg/mL; citric acid, 16,384–16 μg/mL; butyric acid, 16,384–16 μg/mL; and acetic acid, 32,768–32 μg/mL; except for l-lactic acid, 16,384–16 μg/mL. Acetic acid was purchased from EM Science (Gibbstown, NY, USA). Citric, butyric, propionic, and formic acids were purchased from Sigma-Aldrich (Milwaukee, WI, USA). l-Lactic acid was purchased from Alfa Aesar (Wad Hill, MA, USA). Reverse osmosis water was used to dilute the OAs to make standard solutions, and they were filter-sterilized using a 0.2 μm × 25 mm syringe filter (# 431224, Corning Inc., Corning, NY, USA).

### 2.3. Determination of pH in Wells of the 96-Well Microplates at the *C. jejuni* MICs

pH analysis was performed as previously described [52]. Briefly, the pH was determined of the solutions in the MIC wells for all OAs in triplicate, and the means and standard deviations were calculated. The solutions at the same MIC from 16-wells (100 μL/well) for each OA were added together (for a total of 1600 μL) in a 5 mL sterile microtube (Argos Technologies, Inc., Vernon Hills, IL, USA). An Orion benchtop pH meter with a ROSS Ultra glass combination pH electrode was used to determine the pH (Thermo Fisher Scientific, Chelmsford, MA, USA).

### 2.4. The Calculated Ratio of Undissociated/Dissociated OAs

It is well-known that when the pH and pK_a_ are known, the ratio of the undissociated/dissociated weak organic acid concentration can be calculated using the Henderson-Hasselbalch equation [62]:

pH=pKa+log([A−][HA])

where pK_a_ is equal to the −log_10_ of the organic acid dissociation constant (K_a_), [A^–^] is the molar concentration of the conjugate base (dissociated organic acid), and [HA] is the molar concentration of the undissociated organic acid [62]. Rearrangement of the Henderson-Hasselbalch equation can provide the ratio of the undissociated/dissociated weak organic acid [40]:

ratio=[HA][A–]=110pH–pKa

The ratio of the undissociated/dissociated acid can then be calculated using the published pK_a_ of each OA and the results of the measured pH at the MICs. The pK_a_ for propionic, l-lactic, formic, citric, butyric, and acetic acid is 4.87, 3.86, 3.75, 3.14, 4.82, and 4.75, respectively. The pH was experimentally determined, the molar concentrations of the OAs are known at the bacterial MICs, and the molar concentrations of the undissociated and dissociated OA species at each MIC can then be calculated from the ratio obtained from the Henderson-Hasselbalch equation [48,49,50,51,52].

### 2.5. Statistics

The central tendency of the organic acid MICs and MIC_M_s for the 96 *C. jejuni* strains isolated from chicken houses was defined by showing the 90th percentile, range, mode, and median of the data. The mean and standard deviation of triplicate pH samples at each MIC for each OA were calculated.

## 3. Results

### 3.1. Susceptibility Studies of OAs Against the *C. jejuni* strains

Table 1 presents the MICs and MIC_M_s data determined for the 96 *C. jejuni* strains against all the OAs evaluated. l-Lactic and formic acid MIC_M_s demonstrated the greatest levels of OAs needed to inhibit all the *C. jejuni* strains tested (90.94 and 89.0 mM), respectively. The OA molar concentration required for inhibition of *C. jejuni* by these two acids was followed by the molar concentrations of acetic, propionic, and butyric acid of 68.21, 55.29, and 46.49 mM, respectively. The lowest molar concentration required to inhibit the *C. jejuni* strains was for citric acid (21.32 mM). The *C. jejuni* citric acid MIC_M_ concentrations were by far the lowest levels observed of all the OAs tested.

The central tendency of the MIC data is shown in Table 2. The central tendency is a typical value found for a probability distribution. The highest median values are for l-lactic acid, formic acid, and butyric acid of 22.74, 22.25, and 23.24 mM, respectively, while the highest range values are for l-lactic acid, formic acid, acetic acid, and propionic acid of 90.94, 89.00, 68.21, and 55.29 mM, respectively, and the 90th percentile follows the highest range values for l-lactic, formic, and acetic acid of 45.47, 44.50, and 34.10 mM, respectively, except for butyric and propionic acid of 23.25 and 13.82 mM, respectively.

### 3.2. Experimentally Determined pH at the *C. jejuni* MICs for the 96 Strains Evaluated with the OAs

**Figure 1:**
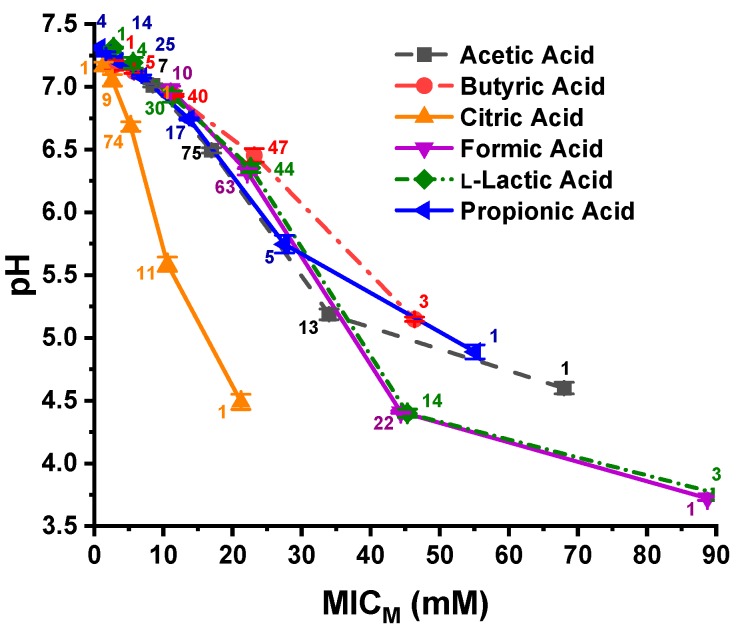
Presentation of pH at the MIC_M_s of propionic, l-lactic, formic, citric, butyric, and acetic acids for 96 *C. jejuni* strains. The number of strains is displayed adjacent to the data points. The pH data points represent the mean and standard deviation of triplicate samples.

The 96 *C. jejuni* strains were grouped together as one group for each OA; therefore, the six OAs resulted in six OA groups, each containing 96 strains. The experimentally determined pH values of the 96-well plates at the *C. jejuni* MIC_M_s are shown in a graphical presentation in Figure 1. Triplicate samples were evaluated at each pH data point, and the number of strains at each MIC_M_ is depicted next to each data point. The pH necessary to inhibit 100% of the strains with butyric acid was 5.14, and that for 99% of the strains with propionic, citric, and acetic acid was 5.74, 5.58, and 5.18, respectively. However, the MIC_M_ pH for 99% of the bacterial strains evaluated with formic acid and 97% of the strains against l-lactic acid was 4.4 and 4.39, respectively. The pH value needed for inhibition of 97–100% of *C. jejuni* against the six OAs falls into two different pH groupings, which has, on average, 1.01 pH unit difference. The pH at 100% inhibition of the *C. jejuni* strains against formic acid and l-lactic acid is 3.71 and 3.74, respectively. Using these values, the pH difference for the two groupings with 100% of the strains being inhibited by formic acid and l-lactic acid is, on average, 1.69 pH units.

### 3.3. Calculated Undissociated OA Concentrations at the *C. jejuni* MIC_M_s

**Figure 2:**
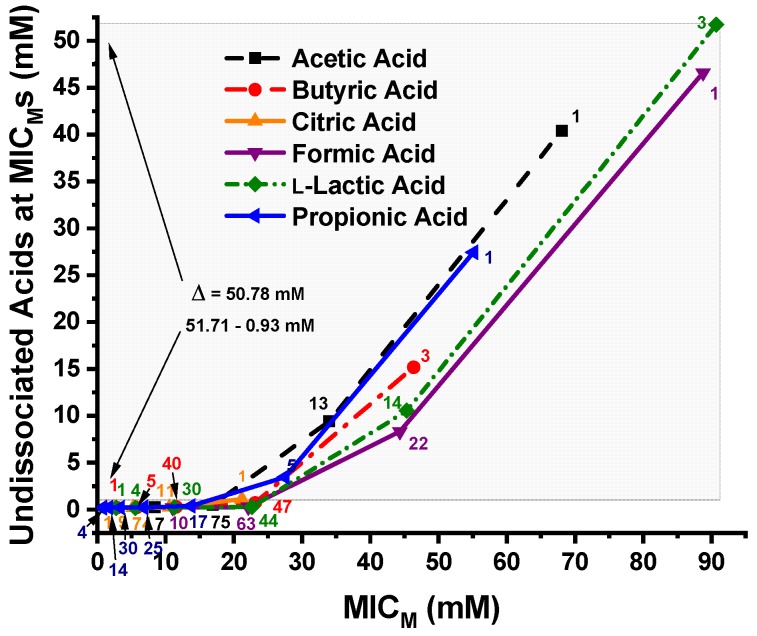
Concentrations (mM) of the six undissociated organic acids—propionic, l-lactic, formic, citric, butyricand acetic— at the MIC_M_s of the 96 *C. jejuni* strains. The large shaded area shows the difference between the undissociated l-lactic acid and citric acid levels required for disinfection of all 96 strains by all organic acids tested; Δ = 50.78 mM. The number of strains is displayed next to each MIC_M_ data point.

The undissociated OA concentrations of propionic, l-lactic, formic, citric, butyric, and acetic acids calculated using the Henderson-Hasselbalch equation at the MIC_M_s of the 96 *C. jejuni* strains are shown in Figure 2. The undissociated l-lactic, formic, acetic, propionic, and butyric acid levels at the MIC_M_ of 100% of the *C. jejuni* strains tested was 51.71, 46.54, 40.31, 27.33, and 15.05 mM, respectively, while the undissociated citric acid concentration of all 96 *C. jejuni* strains at their MIC_M_s was only 0.932 mM. A difference of Δ = 50.78 mM OA between the MIC_M_ of all 96 strains against l-lactic acid and citric acid are as shown by the large shaded area in Figure 2.

**Figure 3:**
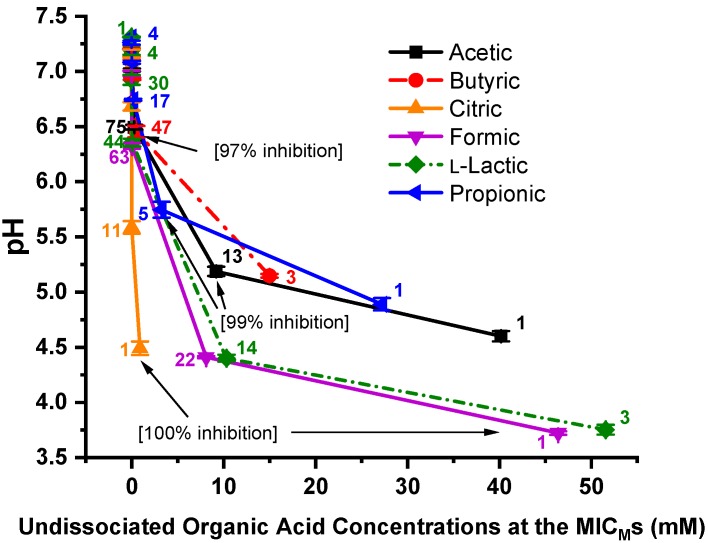
Plot of pH vs. MIC_M_s of the 96 *Campylobacter jejuni* strains against the six undissociated organic acids, propionic, l-lactic, formic, citric, butyric, and acetic acids. The number of strains is displayed adjacent to the MIC_M_ data points. Each pH data point is the mean and standard deviation from triplicate samples.

Figure 3 shows the pH vs. undissociated OA concentrations at the MIC_M_s of the six OAs against the 96 *C. jejuni* strains. The number of *C. jejuni* strains at the MIC_M_ is displayed adjacent to each data point. Most of the *C. jejuni* strains were inhibited by undissociated OAs at concentrations approaching zero. The difference in the points where both citric acid and l-lactic acid inhibit 100% of the strains is approximately 51 mM undissociated OA and a Δ pH = 0.74 unit. At the points where 97% of the strains were inhibited by undissociated butyric acid and all 96 strains were inhibited by undissociated citric acid, the undissociated OA concentrations approach zero and the Δ pH = 1.97 unit. At the points where 99% of the strains were inhibited by undissociated acetic acid and propionic acid, there is approximately a 5 mM difference in undissociated OA concentrations and the Δ pH = 0.56 unit.

### 3.4. Calculated Dissociated OA Concentrations at the *C. jejuni* MIC_M_s

**Figure 4:**
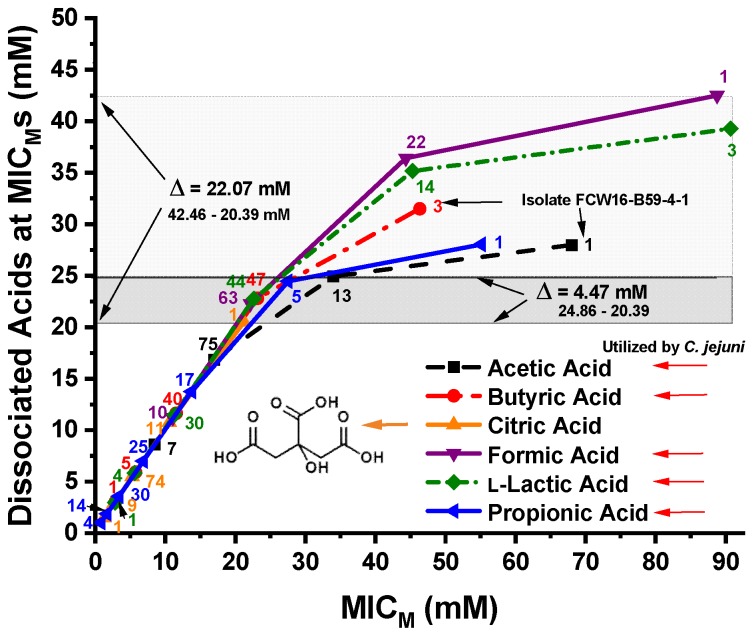
Concentrations (mM) of the six dissociated organic acids—propionic, l-lactic, formic, citric, butyric, and acetic—at the MIC_M_s of the 96 *C. jejuni* strains. The larger, lightly shaded band shows the difference between dissociated citric acid and formic acid concentrations required for inhibition of 100% of the strains for all organic acids, Δ = 22.07 mM, in excess of the level required for inhibition of all strains by citric acid. The line through the citric acid 1-strain (100% of strains) and acetic acid 13-strains (99% of strains) MIC_M_ data points are encompassed by the narrow dark band or difference in acid concentrations for inhibition of 100, 97, 99, and 99% of the *C. jejuni* strains by citric, butyric, acetic, and propionic acids, respectively, Δ = 4.47 mM. The five arrows pointing toward the organic acids on the lower right of the figure are those OAs suggested in the literature as being utilized by *C. jejuni*. The number of strains is displayed adjacent to each MIC_M_ data point.

The dissociated OA concentrations of propionic, l-lactic, formic, citric, butyric, and acetic acids calculated using the Henderson-Hasselbalch equation at the MIC_M_s of the 96 *C. jejuni* strains are shown in Figure 4. The dissociated OA concentrations required to produce MIC_M_s for all 96 *C. jejuni* strains by citric acid and 99% of the strains by propionic acid and acetic acid and 97% of the strains by butyric acid are shown by the narrow, darker-shaded band in Figure 4. This darker-shaded band shows a Δ = 4.47 mM difference between the MIC_M_ of all 96 *C. jejuni* strains inhibited by citric acid, and 99% of the strains inhibited by acetic acid. The MIC_M_ for all 96 strains occurred at a dissociated citric acid level of 20.39 mM, and the MIC_M_ for 95 of 96 strains occurred at a dissociated citric acid concentration of 10.62 mM. The MIC_M_ for 100% of the strains for all dissociated OAs occurred at a level of 42.46 mM formate. However, only the results for citric acid are not affected by *C. jejuni* OA utilization.

**Figure 5:**
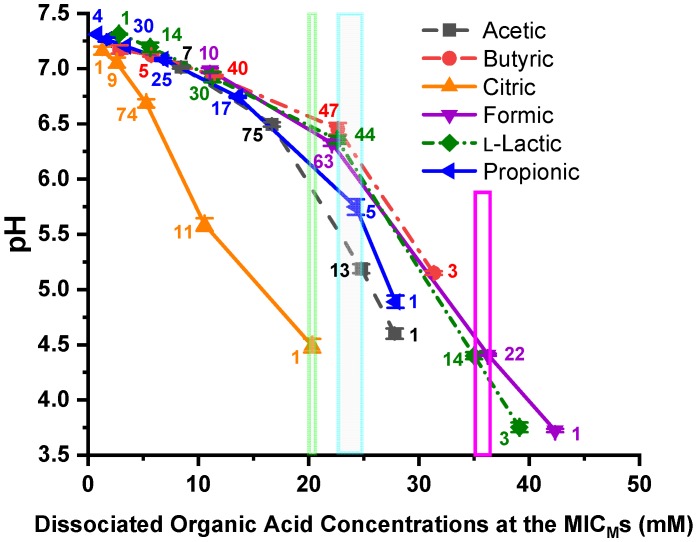
pH vs. MIC_M_s of the 96 *C. jejuni* strains against the six dissociated organic acids—propionic, l-lactic, formic, citric, butyric, and acetic acid. The number of strains is displayed adjacent to each MIC_M_ data point. The small, light green, vertical box at 20.39 mM shows the inhibition of 100% of the strains by dissociated citric acid. The larger light blue vertical box (22.72 to 24.86 mM) encloses the inhibition of 97–99% of the *C. jejuni* strains by dissociated butyric, propionic, and acetic acid, Δ = 2.14 mM. The magenta vertical box (35.11 to 36.36 mM) encloses the inhibition of 97–99% of the *C. jejuni* strains by dissociated l-lactic and propionic acid, Δ = 1.25 mM. Each pH data point is the mean and standard deviation of triplicate samples.

The plot of pH vs. dissociated OA concentrations at the *C. jejuni* MIC_M_s against the six OAs is shown in Figure 5. The number of strains is displayed adjacent to each data point. The narrow, light green vertical box (20.39 mM) depicts the data point for bacterial inhibition of all 96 strains by dissociated citric acid. The larger, light blue vertical box (22.72 to 24.86 mM dissociated OAs) encompasses the data points for bacterial inhibition of 97–99% of the *C. jejuni* strains by dissociated butyric, acetic, and propionic acid. The red box encompasses the data points for bacterial inhibition of 97–99% of the strains by l-lactic acid and formic acid. Each pH data point is the mean and standard deviation of triplicate samples.

## 4. Discussion

Food processing plants often base pathogen decontamination strategies on pH [63,64]. Organic acids are regularly used to remove foodborne bacteria from poultry carcasses in the United States [28,29,30]. However, many foodborne pathogens have acid stress mechanisms that can help them adapt to varying pH environments [38,65,66,67,68]. *Campylobacter jejuni* has the gene make-up required to induce an acid-tolerance response [68]. In addition, there is an increase of acid tolerance in *C. jejuni* caused by coincubation with protozoa [69], and the genes involved in iron control and uptake are also induced [67,68]. We evaluated the interactions of six different OAs—propionic, l-lactic, formic, citric, butyric, and acetic—against 96 *C. jejuni* potential foodborne pathogen strains, while evaluating the effects that pH and the undissociated and dissociated OA species have on the bacteria at their OA MIC_M_s.

The central tendency of the MIC data show that median MIC_M_ values for butyric, l-lactic, and formic acid necessary for inhibition of the same strains have the highest values and the median acetic acid MIC_M_ necessary for inhibition of the *C. jejuni* strains has an intermediate value, while the median MIC_M_ values for inhibition by propionic and citric acid have the lowest values. However, l-lactic acid, followed by formic acid and acetic acid, have the highest ranges of MIC_M_ values, and l-lactic acid and formic acid have the highest 90th percentile values, while citric acid has the lowest overall inhibition concentration (10.66 mM). The citric acid 90th percentile value is the same value as was observed for citric acid in a previous study of *C. coli* [52]. It has previously been shown that citrate cannot be utilized by whole cells of *Vibrio fetus* (*Campylobacter fetus*) as an alternative energy source [70], nor by *C. jejuni* from swine [71]. Our data also suggests that citric acid is not utilized by *C. jejuni*, thereby making citric acid a good candidate to be used for disinfection of this bacterium.

### 4.1. Differences in pH Between Solutions of Different OAs at the MIC_M_

Interestingly, it took a pH of 4.39 and 4.4 to inhibit 97% and 99% of the strains by l-lactic acid and formic acid, respectively, while it took a pH of 6.45, 5.74, 5.58, and 5.18 to inhibit 97%, 99%, 99%, and 99% of the strains by butyric, propionic, citric, and acetic acid, respectively. This is an average of 1.34 pH unit difference between the pH necessary to inhibit 97–99% of the same 96 *C. jejuni* strains by these two groups of acids. We have reported pH differences at the MIC_M_s of other Gram-negative strains against OAs, and only one other bacterium had a greater pH difference between inhibition of all strains, *C. coli* with a 1.76 pH unit difference [52]. The inhibition of 98% of 175 *Pseudomonas aeruginosa* strains by different OAs had a 0.98 pH unit difference [72]. The inhibition of 98% of 344 *E. coli* O157:H7 strains had a 0.56 pH unit difference between three different OAs [48], the inhibition of 138 non-O157 STEC strains had a 0.99 pH unit difference between four different OAs [49], and inhibition of 95% of 145 *Salmonella* strains by four different OAs resulted in an observed 1.1 pH unit difference [50]. These data demonstrate that inhibition of *C. jejuni* or the other Gram-negative bacteria previously studied are primarily not dependent on pH, as others have suggested [40], but bacterial inhibition by OAs must be dependent on some other aspect of the OAs [52,73]. One might expect that the MIC_M_s for the same bacteria against different OAs would be at the same pH value if pH were the primary cause of inhibition [52], but we did not observe that during experimentation.

### 4.2. Relationships of MIC_M_ Concentrations and pH to the Undissociated OA Concentrations

The inhibition of 100% of all 96 *C. jejuni* strains by the six undissociated OAs, propionic, l-lactic, formic, citric, butyric, and acetic acid required concentrations of 0.932 mM citric acid to 51.71 mM l-lactic acid, which is a difference in the level of undissociated acid of 50.78 mM across all six OA species for inhibition of the same 96 strains. The MICs of most of the strains are observed at very dilute undissociated OA concentrations that approach 0.5 to 5 μM. No correlation was observed as to the concentration of undissociated OAs required to produce the MIC_M_s for the 96 *C. jejuni* strains. These results agree with our earlier studies on other Gram-negative foodborne pathogens. Inhibition of 100% of *P. aeruginosa* (175 strains) by undissociated citric (2.53 mM) or undissociated acetic acid (21.65 mM) resulted in a difference of Δ = 19.12 mM OAs [72]. Inhibition of 98.3% of *E. coli* O157:H7 (344 strains) by undissociated citric (2.86 mM) or undissociated acetic acid (50.63 mM) was a difference of Δ = 47.77 mM OAs for disinfection of these strains [48]. Inhibition of 100% of non-O157 STEC (138 strains) by undissociated citric (2.2 mM) and undissociated acetic acid (49.11 mM) resulted in a difference of Δ = 46.91 mM OAs for disinfection of these strains [49]. Inhibition of 100% of *Salmonella* (145 strains) by undissociated citric (2.29 mM) and undissociated acetic acid (19.0 mM) resulted in a difference of Δ = 16.71 mM OAs for disinfection of these strains [50], and inhibition of *C. coli* (111 strains) by undissociated citric (0.024 mM) or undissociated acetic acid (39.93 mM) resulted in a difference of Δ = 39.91 mM [52]. In all five cases, the undissociated OA concentrations did not correlate with the MIC_M_s of the different bacteria. The levels of undissociated acids necessary to inhibit non-O157 STECs, *E. coli* O157:H7, *C. coli*, and in this study for *C. jejuni* were the highest, but most likely this increase was caused because of the glutamate and arginine-dependent acid-resistance systems [66] used to protect *E. coli* strains from extreme acid stress or the maintenance of iron homeostasis to protect against oxidative stress and acid resistance in *Campylobacter* spp. [74] and specifically *C. jejuni* [67,68], or bacteria utilization of OAs as an energy source.

The concentrations of undissociated OAs required to inhibit the 96 strains can be viewed from another perspective. Looking at the pH of the system at the points where the 96 strains are inhibited by the undissociated OA clearly shows that close to all 96 strains are inhibited by undissociated citric acid as the undissociated OA concentration approaches zero, and in fact, most of the strains are inhibited by all six organic acids as the undissociated OA concentrations approach zero. These results are not realistic, suggesting that the undissociated OAs play a small part, if any, in inhibiting *C. jejuni*.

### 4.3. Relationships of MIC_M_ Concentrations and pH to the Dissociated OA Concentrations

The inhibition of 96 *C. jejuni* strains by dissociated citric acid at 20.39 mM and inhibition of 99% of the strains by propionic and acetic acid at 24.36 and 24.86 mM, respectively, and 97% of the strains by butyric acid at 22.72 mM formed a narrow inhibition band with a Δ = 4.47 mM. This small concentration range observed for inhibition of most of the strains (97–99%) is very defined, unlike what was observed for the undissociated acids. However, the largeness of the *C. jejuni* inhibition concentration range against dissociated l-lactic and formic acid is most likely due to the bacterial utilization of these two acids. Formate is metabolized by the multi-subunit formate dehydrogenase (FDH) in *C. jejuni* [75], which exhibits a chemoattraction to and respiration of formate, compared with other OAs making formic acid a primary energy source [76,77]. Other OAs, like acetic [77,78], butyric [77,78], propionic [78], and l-lactic [78,79] can also be utilized by *C. jejuni* as energy sources. *Campylobacter jejuni* utilization of l-lactate proceeds through two NAD-independent l-LDHs—a non-flavin iron-sulfur containing enzyme, and a flavin and iron-sulfur containing enzyme [79]. Therefore, dissociated l-lactic and formic acid may be expected to have a high *C. jejuni* inhibition concentration, as was seen in our experiments. However, it was determined that *C. jejuni* strains isolated from humans did not utilize propionic acid [80]. The susceptibility of *C. jejuni* to OAs has been investigated by several researchers showing inconsistent results [22,31,38,80,81]. Due to *C. jejuni* utilization of acetic, butyric, and propionic acids, our experimental results show increased concentrations of dissociated OAs widening the band of *C. jejuni* inhibition, but the high dependence of *C. jejuni* on formic and l-lactic acid for energy production skews the formic and l-lactic acid curves well beyond those for other OAs. This same dependence on formic acid for energy production was observed in studies with *C. coli* [52], and major utilization of l-lactic acid was observed in a study of *P. aeruginosa* strains [72]. However, a relatively small band (Δ = 5.24 mM) of inhibition resulting from 100% of the strains by l-lactic acid and citric acid and 97–99% of the strains by butyric acid, propionic acid, and acetic acid shows a well-correlated group of dissociated OAs that inhibit *C. jejuni*. Previous studies of *P. aeruginosa* strains resulted in inhibition of 98% of the 175 strains by dissociated citric acid (10.24 mM) and dissociated acetic acid (9.98 mM), resulting in a difference in dissociated OAs of only Δ = 0.26 mM [72]. Inhibition of 98.3% of *E. coli* O157:H7 (344 strains) by dissociated propionic (13.82 mM) and l-lactic acid (19.12 mM) had a difference of Δ = 5.54 mM [48]. In a study of 138 non-O157 STEC strains, inhibition of 100% of the strains was accomplished by dissociated l-lactic acid (12.93 mM) and citric acid (19.12 mM) having a difference of only Δ = 6.19 mM [49]. Inhibition of all 145 *Salmonella* strains by dissociated propionic (13.67 mM) and citric acid (19.03 mM) had a small difference of Δ = 5.36 mM [50], and inhibition of 111 *C. coli* strains by dissociated butyric (22.56 mM) and l-lactic acid (21.17 mM) resulted in a very small difference of Δ = 1.39 mM [52]. However, citric acid was not utilized by *C. coli* [52,71] and inhibition of 100% of the 111 *C. coli* strains by dissociated citric acid occurred at 10.64 mM [52]. It should be noted that 95 of 96 *C. jejuni* strains were inhibited by dissociated citric acid at 10.62 mM in this study.

Alternatively, we can look at the pH vs. the dissociated OA concentrations at the MIC_M_s. The pH varies widely at the *C. jejuni* MIC_M_s against the dissociated OAs, but dissociated citric acid, the only OA not utilized by *C. jejuni* as an energy source, inhibited 100% of the strains at a concentration of 20.39 mM. This dissociated OA value compares well with dissociated OA values obtained from previous studies of many other pathogens. Inhibition of 100% of *Salmonella* strains by all dissociated OAs tested required ≤19.03 mM [50]; all VRE strains were inhibited by all dissociated OAs tested at ≤19.53 mM [51]; all *P. aeruginosa* strains were inhibited by dissociated citric and acetic acid using a level of ≤18.79 mM [72]; all non-O157 STEC strains were inhibited by all dissociated OAs tested at a level of ≤19.12 mM [49]; all *C. coli* strains were inhibited by dissociated citric acid using a level of ≤10.64 mM [52]; and 98.3% of *E. coli* O157:H7 strains were inhibited by all OAs tested using a level of ≤19.36 mM [48]. Approximately 20 mM dissociated OA appears to be an optimum level for inhibition of the bacteria tested, unless the OA is utilized by the bacteria. The inhibition of 97–99% of the strains by dissociated propionic, acetic, and butyric acid are also utilized by *C. jejuni* required levels of ≤24.86 mM [77,78], and have similar OA concentrations at the point of inhibition of the *C. jejuni* strains (Δ = 2.14 mM). The inhibition of 97–99% of the strains by dissociated l-lactic acid and formic acid are utilized in a major way by *C. jejuni* required levels of ≤36.36 mM to inhibit the *C. jejuni* strains. Unlike the undissociated OAs having a broad concentration pattern at the MIC_M_s of the bacteria, the dissociated OAs have similar concentrations when inhibiting 97–100% of the 96 *C. jejuni* strains. The work described here is extremely promising, and provides insight into the mechanism of OA inhibition of bacteria, and also correlates well with our previous work on six other pathogenic bacteria. However, the studies described here were conducted in a laboratory setting under controlled conditions and controlled concentrations of bacteria as well as OAs, and further confirmation work in the field must be performed to confirm the results obtained in the laboratory.

### 4.4. Mechanism by Which Organic Acids Control Bacteria Based on Our Studies

The mechanism of OA bacterial inhibition has traditionally been assumed to be mainly dependent on pH [38] or the undissociated form of the OAs [39,40,41,42,43,44]. The activities of OAs change with pH levels, and the type and concentration of OA [47]; and OAs affect bacteria differently depending on whether the bacteria utilize the OA as an energy source [52]. An underlying premise of previous studies is that the OA must be protonated in order to pass through the outer membrane of the bacterial cell. Our present study with *C. jejuni* clearly shows that dissociated OA levels correlate best with the MIC_M_s of *C. jejuni*, suggesting that the earlier premise followed by researchers might be wrong. Even though *C. jejuni* utilizes many of the OAs studied as energy sources, these dissociated OAs still correlate better with the MIC_M_s than either pH alone or the undissociated OAs. Almost all 96 *C. jejuni* strains (97–100%) were inhibited by either dissociated citric, propionic, acetic, or butyric acid in the concentration range of 20.39–24.86 mM. Formic and lactic acid required much higher levels of dissociated OA for inhibition, most likely because these two OAs are well-utilized by *C. jejuni* as energy sources [76,77,79]. A previous publication states that “intracellular accumulation of anions is a primary contributor to inhibition of bacterial growth” [46], and our previous studies and these *C. jejuni* data fit well with that statement. Also, our previous work on six other pathogens, *E. coli* O157:H7 [48], non-O157 STECs [49], *P. aeruginosa* [72], *Salmonella* [50], VRE [51], and *C. coli* [52] correlates well with this current *C. jejuni* study. The first five bacterial pathogens required a dissociated OA concentration of 12.45–19.76 mM for inhibition of nearly all strains tested, except for lactic acid which is utilized by *P. aeruginosa* as an energy source [72]. *C. coli* requires a dissociated OA concentration of from 10.64–22.56 mM for inhibition by the OAs not utilized as an energy source for *C. coli* [52]. Therefore, we have generated a large data pool that demonstrates bacterial inhibition by OAs is caused by a distinct concentration of the dissociated OA species, and the concentration is similar regardless of what OA is used, except for those OAs that are utilized by the bacteria as energy sources. In accordance with the Henderson-Hasselbalch equation, the pH, pK_a_, and the concentration of OA acid will specifically produce a concentration of dissociated OA species that inhibits bacteria. For the many pathogenic bacteria that we have studied, the general concentration range of dissociated OA species required to inhibit 97–100% of the bacteria is 20 to 25 mM.

## 5. Conclusions

In our study of *C. jejuni* inhibition by OAs, the inhibition of strains was associated with the concentration of dissociated propionic, citric, butyric, and acetic acid, and these OAs correlated with the MIC_M_s of 97–100% of the *C. jejuni* strains tested. Bacterial inhibition was not dependent on pH or on the concentration of the undissociated OAs. Even though *C. jejuni* cannot utilize the citric acid cycle (CAC) to produce energy, *C. jejuni* can utilize some of the CAC intermediates and many OAs to produce energy: propionic, l-lactic, formic, butyric, and acetic acid can all be utilized by *C. jejuni* to produce energy, and l-lactic and formic acid are known to be good energy sources, while *C. jejuni* exhibits enhanced chemoattraction to formate in comparison to other OAs. This may account for the high concentrations of l-lactic and formic acid required to inhibit *C. jejuni* in our studies. Based on how well *C. jejuni* utilizes formate and l-lactate, the use of l-lactic and formic acid would most likely not be appropriate for disinfection or removal of this bacterium. The other OAs utilized by *C. jejuni*, acetic, butyric, and propionic acid, also would not be expected to be good for removing *C. jejuni* from carcasses, because these acids would have the tendency to revive the bacteria at reduced levels of dissociated acid. Dissociated citric acid showed good properties in our studies, inhibiting 100% of the *C. jejuni* strains at a reasonable concentration, and citric acid is not utilized by *C. jejuni*. A concentration of dissociated citric acid (21 mM) could potentially be used to eliminate *C. jejuni* surface bacteria from animal carcasses. If the concentration of dissociated citric acid is reduced from the suggested 21 mM, then *C. jejuni* bacteria may escape disinfection. However, studies must be undertaken to confirm that the suggested concentrations of dissociated organic acids are adequate to remove *C. jejuni* bacteria in the field vs. the laboratory. If the dissociated OA used in a carcass wash is not controlled at the proper concentration, the wash may not provide the expected level of elimination of surface bacteria. Of the six OAs studied here, citric acid is the most efficient at inhibiting *C. jejuni*.

## Figures and Tables

**Table 1:** The determined organic acid MICs and MIC_M_s ^a^ for 96 *Campylobacter jejuni* strains isolated from shoe covers worn in broiler chicken houses.

Organic Acids	MIC (μg/mL)	MIC_M_ (mM)	No. of *C. jejuni* Strains
Acetic Acid	4096	68.21	1
	2048	34.10	13
	1024	17.05	75
	512	8.53	7
Butyric Acid	4096	46.49	3
	2048	23.25	47
	1024	11.62	40
	512	5.81	5
	256	2.91	1
Citric Acid	4096	21.32	1
	2048	10.66	11
	1024	5.33	74
	512	2.67	9
	256	1.33	1
Formic Acid	4096	89.00	1
	2048	44.5	22
	1024	22.25	63
	512	11.12	10
l-Lactic Acid	8192	90.94	3
	4096	45.47	14
	2048	22.74	44
	1024	11.37	30
	512	5.68	4
	256	2.84	1
Propionic Acid	4096	55.29	1
	2048	27.65	5
	1024	13.82	17
	512	6.91	25
	256	3.46	30
	128	1.73	14
	64	0.86	4

^a^ MIC_M_s = Molar MICs; MIC, minimum inhibitory concentration.

**Table 2:** The central tendency of organic acid MICs and MIC_M_s ^a^ for 96 *Campylobacter jejuni* strains isolated from shoe covers worn in broiler chicken houses.

Organic Acid	Median	Mode	Range	90th Percentile
Acetic Acid				
MIC (μg/mL)	1024	1024	512–4096	2048
MIC_M_ (mM)	17.05	17.05	8.53–68.21	34.1
Butyric Acid				
MIC (μg/mL)	2048	2048	256–4096	2048
MIC_M_ (mM)	23.24	23.25	2.91–46.49	23.25
Citric Acid				
MIC (μg/mL)	1024	1024	256–4096	2048
MIC_M_ (mM)	5.33	5.33	1.33–21.32	10.66
Formic Acid				
MIC (μg/mL)	1024	1024	512–4096	2048
MIC_M_ (mM)	22.25	22.25	11.12–89.00	44.5
l-Lactic Acid				
MIC (μg/mL)	2048	2048	256–8192	4096
MIC_M_ (mM)	22.74	22.74	2.84–90.94	45.47
Propionic Acid				
MIC (μg/mL)	256	256	64–4096	1024
MIC_M_ (mM)	3.46	3.46	0.86–55.29	13.82

^a^ MIC_M_s = Molar MICs.
